# Analysis of clinical features, treatment response, and prognosis among 61 elderly newly diagnosed multiple myeloma patients: a single-center report

**DOI:** 10.1186/s12957-015-0649-8

**Published:** 2015-08-07

**Authors:** Na An, Xin Li, Man Shen, Shilun Chen, Zhongxia Huang

**Affiliations:** Multiple myeloma medical center of Beijing, Department of Hematology, Beijing Chao-yang Hospital, Capital Medical University, 5 Jingyuan Road, Shijingshan District, Beijing, 100043 China

**Keywords:** Multiple myeloma, Elderly patients, Bortezomib, Overall survival

## Abstract

**Background:**

We identified the clinical features of 61 cases of multiple myeloma (MM) patients over 65 years and analyzed the treatment and prognosis of the era of new drugs in elderly patients.

**Methods:**

We identified 61 newly diagnosed symptomatic multiple myeloma (NDMM) among elderly Chinese patients more than 65 years old diagnosed from 2006 to 2012.

**Results:**

Of the 205 consecutive MM patients whom we reviewed, 61 (29.76 %) cases were NDMM patients aged more than 65 years and the others were younger than 65 years old. Among them, 40 (65.6 %) cases were in end-stage (ISS stage III); meanwhile, 19 (31.2 %) cases of them had MM with extramedullary plasmacytoma (EMP), observed in 42.1 % patients at diagnosis, and the top three incidence of position were spinal canal, pleural, and soft tissue. In the whole column, the median follow-up was 38 months and median age was 72.5 years. Patients received bortezomib- or thalidomide-containing regimens as initial therapy. Comparing the two treatment groups, the complete remission (CR)/near-complete remission (nCR) rate was significantly higher in the bortezomib-containing regimens (61.5 vs.18.18 %, *P* = 0.001), no difference in progression-free survival (PFS) and overall survival (OS). Patients of age over 75 years had shorter OS than those of age over 65 years (49 vs. 24 months, *P* = 0.001). The patients with EMP had shorter OS than those without EMP in two age groups (32 vs. 42 and 15 vs. 24 months, *P* = 0.017 and 0.024, respectively).

**Conclusions:**

The results highlight that patients over 75 years and MM with EMP have a poorer outcome. While the CR rate is higher in bortezomib-containing regimens, no significant improvement is noted in respect to the survival outcomes; also, it cannot overcome the negative influence on survival of age and MM with EMP in elderly patients.

## Background

Multiple myeloma (MM) is still an incurable disease, representing the second most common hematologic malignancy worldwide [1]. In Asian countries, there is growing evidence that recognition of MM is increasing rapidly, doubling of MM incidence in the last 10 years [2–4]. In recent years, application of new drugs improves the prognosis of MM patients, including the elderly [5]. However, old patients often present with comorbid conditions, which may decrease their ability to tolerate myelosuppressive regimens; age-related survival inequality was also observed [6]. Currently, limited information is available on the treatment of newly diagnosed symptomatic multiple myeloma (NDMM) among elderly Chinese patients. In this paper, initial treatment response and survival of 61 cases of NDMM in elderly Chinese patients are analyzed. This represents one of the larger series examining the outcomes of elderly patients with NDMM of a single center report from China in recent years.

## Methods

The clinical data of 61 cases of NDMM elderly patients had been diagnosed from Beijing Chao-Yang Hospital (western campus) from March 2006 to March 2012. They were divided into two groups as age: group A (age ≥65, 65–74 years) and group B (age ≥75 years). The diagnosis was based on the International Myeloma Working Group (IMWG) diagnostic criteria. Patients who had an organ involvement with light-chain amyloidosis at the time of diagnosis were not included in the current analysis. MM patients with extramedullary plasmacytoma (EMP) was observed in 42.1 % patients at diagnosis and 57.9 % patients during the disease course, detected by magnetic resonance imaging (MRI), computed tomography (CT), and/or histopathological analysis of biopsy specimens.

In our study, patients received initial therapy with regimens containing bortezomib or thalidomide, for 1–8 cycles. The mean duration of bortezomib-containing regimens was 4 cycles. In patients achieving a complete response (CR) or very good partial response (VGPR), the regimen was repeated for 2–4 cycles as consolidation therapy. In the absence of this, the treatment regimen was modified. Maintenance therapy when utilized was mostly with thalidomide 100 mg/day; the other maintenance regimens included small doses of corticosteroids or lenalidomide. The patients who were unable to tolerate the side effects or were in poor physical condition did not receive maintenance therapy.

Aspirin or low molecular weight heparin to prevent venous thrombosis was routinely used in patients without contraindications. Herpes zoster prophylaxis was routinely used while patients were on bortezomib. Bisphosphonates were used intravenously for myeloma bone prophylaxis monthly.

In this elderly cohort, the patients who were diagnosed as plasma cell leukemic and undergo autotransplant were specifically excluded. Lenalidomide is approved on sale in China in June 2013, but it has not been approved for treatment with the newly diagnosed myeloma patients. So in the current study, the patients received lenalidomide only as maintenance.

The patients were assessed after the completion of 2 cycles of chemotherapy. Responses were graded using the IMWG Uniform Response Criteria, and adverse events were assessed according to the National Cancer Institute Common Terminology Criteria for Adverse Events (CTCAE) version 3.0.

### Ethics statement

The study was approved by the Ethics Committee of Beijing Chao-yang Hospital, Capital Medical University, and follow-up information was obtained with consent of the patients. All aspects of the study were conducted in accordance with the principles of the Declaration of Helsinki.

We used SPSS19.0 (SPSS Inc., Chicago, IL, USA) statistical software for data analysis, and the Kaplan-Meier method was used to assess overall survival (OS) and progression-free survival (PFS). *P* < 0.05 (log-rank test) was considered statistically significant.

## Results

Of all the 61 cases of patients, the median age at diagnosis was 72.5 years. The male and the female were 62.3 % (38/61) and 37.7 % (23/61) cases, respectively. In terms of International Staging (ISS), 65.6 % (40/61) and 32.8 % (20/61) and 1.6 % (1/61) cases were at stage III, II, and I, respectively. Among them, 31.2 % (19/61) cases were diagnosed as MM with EMP, and the top three incidence of position were spinal canal, pleural, and soft tissue. The median follow-up was 38 months (24–96) at the last follow-up. The general clinical characteristics were provided in Tables 1, 2, and 3.

**Table 1:** General clinical characteristics of 61 cases of elderly NDMM patients

Characteristics	Overall	Groups	
Age (years)	72.5 (*n* = 61)	A (≥65, *n* = 37)	B (≥75, *n* = 24)
Gender			
Male	38 (62.3 %)	23 (62.2 %)	14 (37.8)
Female	23 (37.7 %)	14 (37.8 %)	10 (41.7)
Calcium >11 mg/dl	4 (6.6 %)	2 (5.4 %)	2 (8.3 %)
Creatinine >2.0 mg/dl	12 (19.67 %)	7 (18.9 %)	5 (20.8 %)
Hemoglobin <10 g//L	48 (78.7 %)	28 (75.7 %)	20 (83.3 %)
Bone lesions	58 (95.1 %)	35 (94.6 %)	23 (95.8 %)
M component at diagnosis			
Ig G	29 (47.5 %)	16 (43.2 %)	13 (54.2 %)
Ig A	16 (26.2 %)	11 (29.7 %)	5 (20.8 %)
Ig D	2 (3.3 %)	2 (5.4 %)	0
Light chain	13 (21.3 %)	8 (21.6 %)	5 (20.8 %)
Non-secretory	1 (1.6 %)	0	1 (4.2 %)
Stage of ISS			
I	1 (1.6 %)	0	1 (4.2 %)
II	20 (32.8 %)	13 (35.1 %)	7 (29.2 %)
III	40 (65.6 %)	24 (64.9 %)	16 (66.7 %)
MM with EMP	19 (31.2 %)	12 (32.4 %)	7 (29.2 %)
Abnormal genetics testing			
G-banded karyotype testing	4 (6.6 %)	3 (11.5 %)	1 (3.8 %)
FISH	13 (21.3 %)	7 (26.9 %)	5 (19.2 %)

**Table 2:** Clinical characteristics of MM with EMP

Characteristics	Overall (*n* = 19)	A (≥65)	B (≥75)
Age	72 (65–81)	69 (65–73)	77 (75–81)
Gender, *n* (%)			
Male	10 (52.6 %)	7 (36.8 %)	3 (15.8 %)
Female	9 (47.4 %)	5 (26.3 %)	4 (21.1 %)
Heavy chain, *n* (%)			
IgG	9 (47.4 %)	6 (31.6 %)	3 (15.8 %)
IgA	6 (31.6 %)	3 (15.8 %)	3 (15.8 %)
Light chain, *n* (%)			
κ light chain	1 (5.3 %)	1 (5.3 %)	0
λ light chain	2 (10.6 %)	1 (5.3 %)	1 (5.3 %)
β2MG ≥ 5.5 mg/l, *n* (%)	14 (73.7 %)	10 (52.6 %)	4 (21.1 %)
Sites of EMP, *n* (%)			
1. Spinal canal	10 (52.6 %)	8 (42.1 %)	2 (10.5 %)
2. Pleura	5 (26.3 %)	0	5 (26.3 %)
3. Soft tissue	2 (10.5 %)	2 (10.5 %)	0
More than two sites, *n* (%)	4 (21.1 %)	4 (21.1 %)	0
Plasma cell percentage, *n* (%)	37.2 (2–91)	37 (2–91)	37.5 (18.5–46.5)
Abnormal genetics testing, *n* (%)			
G-banded karyotype testing	2 (33.3 %)	1 (16.7 %)	1 (16.7 %)
FISH	4 (66.7 %)	0	4 (66.7 %)

**Table 3:** Genetics test results of the elderly NDLM patients

Patient No.	Chromosome	FISH
1	13 q14 and 14q32	No FISH
2	46xx	Amplification of 1q21
3	46xx	IGH/FGFR3+
4	46xx	Negative
5	46xx	Amplification of 1q21
6	46xy	Amplification of 1q21, IGH/FGFR3+
7	46xy	Negative
8	46xx	17p-
9	46xy	Negative
10	46xx	Amplification of 1q21 and CCND1
11	Complex karyotype abnormalities	Negative
12	46xy	17p-, amplification of IGH
13	46xy	Amplification of 1q21
14	46xy	Amplification of 1q21, I GH and FGFR3
15	46xy	No FISH
16	46xy	No FISH
17	46xy	Negative
18	46xx	No FISH
19	46xx	Amplification of 1q21
20	46xx	No FISH
21	46xy	Amplification of 1q21 and IGH/MAF+
22	46xy	Negative
23	Hypodiploid of chromosome abnormalities	17p-, 13q-, and amplification of IGH
24	46xy	Negative
25	Complex chromosome abnormalities	Amplification of 1q21, 17p-, IGH/CCND1+

Twenty-five cases of patients with a chromosome examination, including four cases of abnormal chromosomal changes. Nineteen patients had FISH testing; among them, 13 patients had abnormal FISH changes

Patients received bortezomib-containing regimens as follows: bortezomib (Velcade, V or P) and dexamethasone (D) or VD with cyclophosphamide (VCD) or with adriamycin (A) (PAD). Patients received thalidomide-containing regimens as follows: TAD or melphalan (M) and prednisone (P) thalidomide (T) (MPT) and CTD. Among the above regimens, bortezomib (1.0–1.3 mg/m^2^) was given intravenously or subcutaneously on days 1, 4, 8, and 11 of a 21-day/cycle or weekly and dexamethasone 10–20 mg/day on days 1, 4, 8, and 11/cycle or weekly, cyclophosphamide (200 mg/m^2^) and adriamycin (9 mg/m^2^). Melphalan (4 mg/m^2^/day) and prednisone (40 mg/m^2^/day) were administered orally on days 1–7, PO. Among CTD regimen, cyclophosphamide was given on days 1–4/15–18 intravenously and dexamethasone 10–20 mg/day on days 1–4/15–18, PO, and thalidomide (100 mg) was administered orally each day. In this study, treatment regimens were heterogeneous, but VD or TAD was the most commonly used.

### Adverse events

Adverse events (AEs) of two treatment groups were shown in Table 4; the top three were gastrointestinal symptoms, infection, and peripheral neuropathy. 1–2 grades and tolerated gastrointestinal reactions were found in 30 cases; these included nausea, vomiting, bloating, constipation, diarrhea, and anorexia, of 23 (60 %) cases occurred in the bortezomib-containing regimen groups. All 26 cases suffered infection, most of which were lung bacterial or/and fungal infection, of 14 (63.6 %) cases occurred in the thalidomide-containing regimens groups, especially the patients application of TAD regimen treatment. Eighteen cases suffered 1–2 grades of peripheral neuropathy, and its incidence was similar in both treatment groups of approximately 30 %. These included numbness in hand and foot. The number of patients who suffered thrombocytopenia, elevated blood glucose (among them three cases had steroid diabetes because of glucocorticoid), and thrombosis were 12, 10, and 3. The others included herpes zoster infection of grades 3–4 (two cases), arrhythmia (two cases), and rash (two cases).

**Table 4:** Adverse events (AEs)

Adverse events (AEs)	Bortezomib-containing regimens	Thalidomide-containing regimens
Case (%)	(*n* = 39)	(*n* = 22)
Gastrointestinal symptoms	23 (60 %)	7 (31.8 %)
Infection (bacterial or/and fungal)	12 (30.8 %)	14 (63.6 %)
Peripheral neuropathy	12 (30.8 %)	6 (27.3 %)
Cytopenia	8 (20.5 %)	4 (18.2 %)
Hyperglycemia	5 (12.8 %)	5 (22.7 %)
Thrombosis	0	3 (13.6 %)
Herpes zoster	2 (5.1 %)	0
Arrhythmia	1 (2.6 %)	1 (4.6 %)
Rash	1 (2.6 %)	1 (4.6 %)

### Treatment response

Comparing two treatment groups, the ORR of bortezomib-containing regimens was 94.9 % (37/39) and CR/nCR was 61.5 % (24/39). For the thalidomide-containing regimens, its ORR was 86.4 % (19/22) and CR/nCR rate was 18.2 % (4/22); there were significant difference (*P* = 0.001) in CR/nCR and no significant difference in ORR (Table 5).

**Table 5:** Comparison of efficacy of the two treatment options groups

Treatment options	Cases	ORR%	CR/n CR%
Bortezomib-containing regimens	39	94.9	61.51
Thalidomide-containing regimens	22	86.4	18.2
*P* value		*P* > 0.05	*P* = 0.001

Comparison of bortezomib-containing regimens and thalidomide-containing regimens treatment options. There were significant difference (*P* = 0.001) in CR/nCR and no significant difference in ORR

For two age groups, overall response rate (ORR) was 94.6 % (35/37) and CR/nCR rate was 48.7 % (18/37) in group A. ORR was 87.5 % (21/24) and CR/nCR rate was 41.7 % in group B; there was no significant difference between the two of them.

Of 42.2 % (8/19) cases of patients who suffered MM with EMP at diagnosis, seven cases received bortezomib-containing regimen treatment; their ORR was 85.5 % and CR was 28.6 %.

### Survival outcomes

**Fig. 1:**
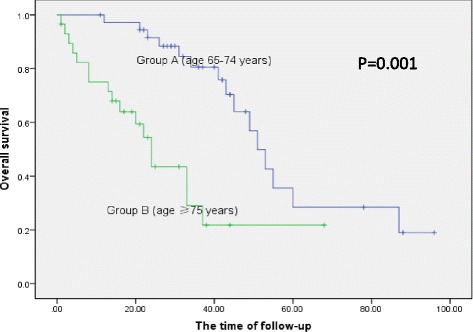
Survival curves in two age groups. There was significant difference in OS between two age groups (*P* = 0.001) but no difference in PFS

Between the two age groups, OS was 37 and 19 months and the median PFS was 22 and 14.5 months, respectively, for group A and group B. There was a significant difference in OS (*P* = 0.001) but no difference in PFS (Fig. 1). OS rates at 1, 2, 3, 4, and 5 years were 82, 73, 60, 35, and 28 %, respectively, in group A. Meanwhile, OS rates at 1, 2, 3, years were 54, 30, and 20 %, respectively, in group B.

**Fig. 2:**
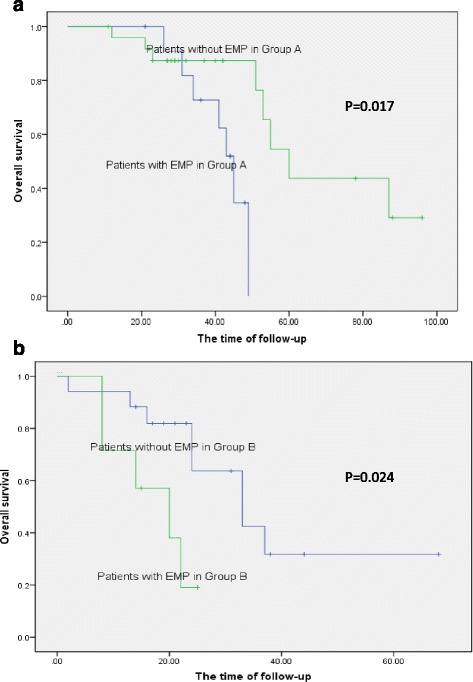
Survival curves in regard to EMP in two age groups. **a** OS of group A in regard to EMP. **b** OS of Group B in regard to EMP. The patients with EMP had shorter OS than patients without EMP in two age groups A and B (*P* = 0.017 and 0.024), respectively

The patients with EMP had shorter OS than those without EMP in two age groups; their OS was 32 vs. 42 in group A and 15 vs. 24 months in group B (*P* = 0.017 and 0.024), respectively (Fig. 2a, b).

Comparing the two treatment options of bortezomib-containing and thalidomide-containing regimens in group A, OS was 41 vs. 36.5 months and PFS was 23 vs. 16.5. So did in group B; OS was 24 vs. 19 months and PFS was 16 vs. 11 months. There were no differences in these two treatment options for OS and PFS.

## Discussion

Of the 205 consecutive MM patients whom we reviewed, 61 (29.76 %) cases were NDMM patients aged more than 65 years and the others were younger than 65 years old. Among them, 40 (65.6 %) cases were in end-stage (ISS stage III) and 19 (31.2 %) cases of them had MM with EMP. Our results are consistent with other reports from Chinese patients; the median age of onset and incidence of MM with EMP are different from the western countries although there have been no accurate estimates of incidence of MM in China [2, 4, 7–10].

In the current study, comparing the bortezomib-containing regimens and thalidomide-containing regimens, the rate and degree of remission all favored the efficacy of bortezomib [5, 10–17], regardless of the age.

Nevertheless, further analysis found that age and MM with EMP had a negative impact on survival. Therefore, the high remission rate benefited by bortezomib did not transfer into a survival advantage. It can be seen among patients ≥75 years, whose PFS and OS were worse compared to those over 65 years, such results clearly indicated greater influence on the survival of the elderly patients, especially the patients of age over 75 years [6, 15]. For patients with EMP, either two different age groups, whose OS was worse with EMP or infiltration of extramedullary patients, further confirmed MM with EMP adversely affect the prognosis [7, 18].

There was no significant survival benefit in older NDMM patients on protease inhibitor therapy in this study, which was inconsistent with other investigators [16–19]. Moreover, the remission rate with bortezomib was not consistent with the OS in our study. One possible explanation for such differences is the fact that the patients of the current study came from a myeloma medical research center and most of the patients were end-stage or with many other poor prognostic factors, such as with higher incidence of EMP. An alternative explanation is that elderly patients have poor tolerance to chemotherapy; in addition, poor compliance also contributes interruption of treatment or inability to adhere to maintenance therapy.

## Conclusions

In general, the current results provide evidence that bortezomib-containing regimens has a deeper response than thalidomide regimens in elderly MM patients, especially in patients of age older than 75 years; however, it cannot overcome the negative influence on survival of age and MM with EMP.

### Consent

Written informed consent was obtained from the patient for the publication of this report and any accompanying images.

## Abbreviations

AEs: adverse events
CR/nCR: complete remission/near-complete remission
CT: computed tomography
DS: Durie-Salmon staging system
EMP: extramedullary plasmacytoma
FISH: fluorescence in situ hybridization
Ig: immunoglobulin
ISS: International staging system
MM: multiple myeloma
MRI: magnetic resonance imaging
ORR: overall response rate
OS: overall survival
PFS: progression-free survival
PR: partial remission
